# Prediction of Spontaneous Breathing Trial Outcome in Critically Ill-Ventilated Patients Using Deep Learning: Development and Verification Study

**DOI:** 10.2196/64592

**Published:** 2025-05-21

**Authors:** Hui-Chiao Yang, Angelica Te-Hui Hao, Shih-Chia Liu, Yu-Cheng Chang, Yao-Te Tsai, Shao-Jen Weng, Ming-Cheng Chan, Chen-Yu Wang, Yeong-Yuh Xu

**Affiliations:** 1 Department of Chest Medicine Division of Respiratory Therapy Taichung Veterans General Hospital Taichung Taiwan; 2 Department of Nursing Hungkuang University Taichung Taiwan; 3 Department of Information Management National Central University Taoyuan Taiwan; 4 Department of Industrial Engineering and Enterprise Information Tunghai University Taichung Taiwan; 5 Department of Computer and Communications Center Taichung Veterans General Hospital Taichung Taiwan; 6 Department of Information Management National Kaohsiung University of Science and Technology Kaohsiung Taiwan; 7 Department of Critical Care Medicine Taichung Veterans General Hospital Taichung Taiwan; 8 Department of Artificial Intelligence and Computer Engineering National Chin-Yi University of Technology Taichung Taiwan

**Keywords:** ventilator, ventilator weaning, respiratory therapy, intensive care unit, artificial intelligence, deep learning, neural networks, spontaneous breathing trial

## Abstract

**Background:**

Long-term ventilator-dependent patients often face problems such as decreased quality of life, increased mortality, and increased medical costs. Respiratory therapists must perform complex and time-consuming ventilator weaning assessments, which typically take 48-72 hours. Traditional disengagement methods rely on manual evaluation and are susceptible to subjectivity, human errors, and low efficiency.

**Objective:**

This study aims to develop an artificial intelligence–based prediction model to predict whether a patient can successfully pass a spontaneous breathing trial (SBT) using the patient’s clinical data collected before SBT initiation. Instead of comparing different SBT strategies or analyzing their impact on extubation success, this study focused on establishing a data-driven approach under a fixed SBT strategy to provide an objective and efficient assessment tool. Through this model, we aim to enhance the accuracy and efficiency of ventilator weaning assessments, reduce unnecessary SBT attempts, optimize intensive care unit resource usage, and ultimately improve the quality of care for ventilator-dependent patients.

**Methods:**

This study used a retrospective cohort study and developed a novel deep learning architecture, hybrid CNN-MLP (convolutional neural network–multilayer perceptron), for analysis. Unlike the traditional CNN-MLP classification method, hybrid CNN-MLP performs feature learning and fusion by interleaving CNN and MLP layers so that data features can be extracted and integrated at different levels, thereby improving the flexibility and prediction accuracy of the model. The study participants were patients aged 20 years or older hospitalized in the intensive care unit of a medical center in central Taiwan between January 1, 2016, and December 31, 2022. A total of 3686 patients were included in the study, and 6536 pre-SBT clinical records were collected before each SBT of these patients, of which 3268 passed the SBT and 3268 failed.

**Results:**

The model performed well in predicting SBT outcomes. The training dataset’s precision is 99.3% (2443/2460 records), recall is 93.5% (2443/2614 records), specificity is 99.3% (2597/2614 records), and *F*_1_-score is 0.963. In the test dataset, the model maintains accuracy with a precision of 89.2% (561/629 records), a recall of 85.8% (561/654 records), a specificity of 89.6% (586/654 records), and an *F*_1_-score of 0.875. These results confirm the reliability of the model and its potential for clinical application.

**Conclusions:**

This study successfully developed a deep learning–based SBT prediction model that can be used as an objective and efficient ventilator weaning assessment tool. The model's performance shows that it can be integrated into clinical workflow, improve the quality of patient care, and reduce ventilator dependence, which is an important step in improving the effectiveness of respiratory therapy.

## Introduction

According to statistics from the National Health Insurance Administration, Taiwan saw an increase of 175,148 patients dependent on ventilators in 2017, with 70% of intensive care unit (ICU) patients requiring ventilator support. This significant trend in ventilator usage persists in Taiwan, irrespective of its impact on ongoing health crises. The current medical practice requirements involving ventilator use include initiating ventilator support, conducting spontaneous breathing tests, and assessing readiness for ventilator weaning. In practical terms, respiratory therapists must perform a ventilator weaning assessment before a spontaneous breathing trial (SBT). This process is complex, meticulous, time-consuming, and spans a period of 48-72 hours. In other words, successful weaning from a ventilator for patients depends on passing an SBT.

In retrospective studies [1,2], confirming whether all patients receive the same SBT strategy is essential based on the specific study design. SBT strategies typically include fixed pressure support (PS) and positive end-expiratory pressure (PEEP). In this paper, the study's SBT strategy was set at a PS of 10 cm H_2_O and a PEEP of 5 cm H_2_O, lasting 2 hours. A successful SBT is generally defined as the patient's ability to maintain a stable respiratory state during the trial without mechanical ventilation support and be safely extubated at the end of the trial. An unsuccessful SBT is defined as a patient experiencing respiratory distress, hypoxemia, hypercapnia, or other conditions requiring the reinitiation of mechanical ventilation during the trial.

Conventional weaning criteria often involve manual selection, with therapists assessing patients based on their responses to a ventilator weaning assessment form to determine if they are eligible for an SBT. However, previous studies have explored the use of information systems to automate ventilator weaning assessments, revealing that this approach can reduce ventilator usage time and enhance the efficiency of respiratory therapists [3]. Research also suggests that automated weaning protocols may decrease the weaning period, theoretically surpassing the need for manual weaning [4,5]. Studies have investigated consciousness status, oxygenation, ventilation modes, and airway protection strategies [6]. Reducing ventilator usage time, weaning time, and ICU stay has been discussed [7]. At the same time, certain elements that cannot be automated in the weaning process have been pointed out [8]. Daily screening methods have effectively decreased dependence on ventilators [9].

Recently, numerous studies have investigated the impact of SBT, including assessing the relationship between end-expiratory lung volumes during SBT and successful ventilator weaning [10]. Some studies have suggested considering respiratory rate (RR) and ventilation time in patients intubated in the ICU for more than 72 hours before extubation after passing an SBT [11]. In different populations, research has cautioned against using an SBT to assess the appropriate timing of extubation for premature infants [12]. Additionally, studies have proposed methods for evaluating extubation in neonates through minimal pressure SBTs [13], by measuring diaphragmatic electrical activity in premature infants to predict extubation success [14], and investigating optimal duration and reasons for failure in pediatric SBTs, noting that a 30-minute SBT may be too brief for pediatric acute respiratory distress syndrome patients in the recovery phase [15]. The impact of SBT on extubation success and prognosis in patients diagnosed with acute exacerbation of chronic obstructive pulmonary disease has been evaluated [16]. On another note, some studies have used oxygen saturation as a crucial indicator for predicting SBT success or failure [17].

However, the factors affecting SBTs and ventilator weaning are diverse and include age [18-20], weaning prediction parameters, sputum volume, cough strength, and consciousness status [21-23], as well as ventilator usage time, disease severity (acute physiology and chronic health evaluation II), and patient nutritional status. Patients experiencing difficult or delayed weaning are often presented with a combination of factors, thus requiring careful assessment by health care professionals. Due to the complexity of patient weaning from ventilators, numerous studies have explored various aspects. For instance, the impact of adding different doses of sedatives (ketamine) on spontaneous breathing patients [24], the correlation between microcirculation during SBTs and successful extubation [25], and for patients passing an SBT, a spontaneous awakening trial could serve as a predictive factor for extubation on the same day. It has also been noted that a higher Richmond Agitation Sedation Scale, no sedative administration on the previous day, no infections, and the absence of neurological diseases or hemodynamic instability increase the chances of extubation on the same day [26].

Using varied SBT strategies can affect outcomes in different studies [27,28]. These differences primarily manifest in several aspects, such as the level of PS, PEEP settings, continuous positive airway pressure (CPAP) use, and the T-piece trial. The level of PS directly impacts the patient's ability to breathe spontaneously. Higher PS, such as 10 cm H_2_O, reduces the patient's breathing effort, potentially leading to a higher SBT success rate. However, this may not fully reflect the patient's breathing capacity at lower support levels. The setting of PEEP affects alveolar stability and airway pressure. Different PEEP settings, such as 5 cm H_2_O, can have varying impacts on a patient's oxygenation and respiratory dynamics, thereby altering the outcomes of the SBT. CPAP provides continuous positive PS, helping to reduce the work of breathing. Conducting an SBT with CPAP at 5 cm H_2_O may make it easier for patients to pass the trial. However, compared to PS strategies, this might offer a different assessment of the patient's respiratory muscle function. The T-piece trial provides no PS, relying entirely on the patient's spontaneous breathing ability. This strategy more accurately reflects the patient's autonomous breathing capacity but may result in a lower SBT success rate due to higher demands on the patient.

Given these differences, it is crucial to recognize how varying SBT strategies can influence the interpretation and comparison of study outcomes. The choice of SBT strategy can significantly affect clinical decisions and patient management. Therefore, understanding the potential impacts of different strategies is essential for improving patient care and optimizing treatment protocols.

Different SBT strategies will lead to varying SBT success rates. Higher PS or the use of CPAP might result in higher success rates, while the T-piece trial might lead to lower success rates. Variations in SBT strategy might also affect postextubation outcomes. If an SBT strategy makes it easier for patients to pass the trial, it could increase the risk of postextubation respiratory distress or the need for reintubation. Due to the different SBT strategies used in various studies, caution is needed when directly comparing study results. The potential impacts of strategy differences on outcomes should be considered.

Artificial intelligence (AI) integrated with health care aims to predict disease diagnosis or prevention through medical alerts. In the current trend of AI applications, incorporating physician expertise into the training of predictive models can provide appropriate treatment for high-risk patients [29]. AI applications in health care include medical robots, AI intelligent diagnosis and image recognition, intelligent drug development, and intelligent health management. Intelligent health management applies AI technology to daily health management, focusing on risk identification, virtual nurses, mental health, web-based consultations, and precision medicine-based health management. Certain studies have used deep learning combined with random forest for risk prediction. One study constructed and validated machine learning models to predict unplanned extubation in ICU patients using electronic health record (EHR) data [30]. Another study developed machine learning algorithms to predict the likelihood of successful weaning from mechanical ventilation using clinical and laboratory data obtained before or shortly after intubation [31]. Recent developments have also explored using reinforcement learning to optimize ventilator management. Using real-world ICU data, a reinforcement learning–based model, EZ-Vent, was proposed to dynamically adjust ventilator settings such as PEEP, FiO_2_ (fraction of inspired oxygen), and tidal volume [32]. A broader review further emphasized the potential of reinforcement learning as a clinical decision support tool in the ICU, highlighting its application in optimizing mechanical ventilation and predicting outcomes related to SBTs [33]. By way of explanation, the introduction of AI can process more significant amounts of data faster, achieve higher accuracy, and assist in decision-making without medical staff experiencing fatigue, thereby implementing intelligent health care models.

The main objective of this study was to develop an AI-based prediction model to predict the outcome of an SBT under a specific SBT strategy using a pre-SBT clinical record gathered just before a patient's SBT. The proposed model is trained and validated based on pre-SBT clinical records to ensure that the model can predict whether an SBT will succeed under a fixed SBT strategy. The proposed model can provide clinicians with an objective and reliable decision-making aid to reduce the subjectivity and errors of traditional manual judgment. The ultimate goal is to facilitate timely and effective clinical decision-making, optimize the weaning process from the ventilator, and improve ICU management efficiency.

## Methods

### Data Source

The respiratory information system database of Taichung Veterans General Hospital served as the data source for this study. We covered the research period from January 1, 2016, to December 31, 2022. We conducted a retrospective case review, including patients aged 20 and older admitted to the ICU during this period. The data were accessed on April 7, 2023, for research purposes. The clinical data were collected from 3686 patients, comprising 6536 pre-SBT clinical records gathered just before each SBT of patients. Among these records, 3268 passed the SBT, while the other 3268 did not pass. Each clinical record includes 2 demographic variables, 7 respiratory parameters, and 5 vital signs, as detailed below (Textbox 1).

The pre-SBT clinical records serve as input features for model training and prediction. These input features comprehensively represent the patient's respiratory and physiological status, providing critical information for the model to assess SBT outcomes effectively. In total, 80% (5228/6536 records) were allocated for training, while the remaining 20% (1308/6536 records) were reserved for testing. In addition, the division process of the training and test datasets follows a random split strategy to ensure that the proportions of different categories (those who passed and failed SBT) remain balanced to avoid bias in the model.

**Details of clinical record.**

- Age—Patient’s age
- Gender—Patient’s gender
- FiO_2_ (%)—Fraction of inspired oxygen, indicating the percentage of oxygen the patient is inhaling
- PaO_2_ (mm Hg)—Partial pressure of oxygen in arterial blood
- VTe (exhaled VT) (mL)—Exhaled tidal volume per breath
- Measured respiratory rate (bpm)—Measured respiratory rate, defined as breaths per minute
- Paw (cm H_2_O)—Peak airway pressure, the highest pressure reached during inhalation
- MAP (cm H_2_O)—Mean airway pressure, representing the average pressure in the airways over the respiratory cycle
- MVe (exhaled MV)—Minute ventilation, the total volume of air exhaled per minute
- Heart rate (bpm)—Number of heart beats per minute
- Systolic blood pressure (mm Hg)—Blood pressure during heart contraction
- Diastolic blood pressure (mm Hg)—Blood pressure during heart relaxation
- SpO_2_ (%)—Oxygen saturation, indicating the percentage of oxygenated hemoglobin in the blood
- Body temperature (°C)—Core body temperature of the patient

### Ethical Considerations

This study was reviewed and approved by the institutional review board (IRB) of Taichung Veterans General Hospital (IRB number: CE23123A), in accordance with institutional and national ethical standards for human subjects research.

The study involved a retrospective analysis of clinical data. All patient data were fully anonymized prior to access and analysis to ensure privacy and confidentiality.

Given the study’s retrospective nature and the anonymization of all data, the IRB waived the requirement for informed consent.

No compensation was provided, as no direct contact with participants occurred, and no new data were collected.

### Spontaneous Breathing Test and Ventilator Weaning Assessment

The successful determination of an SBT was defined as a patient undergoing a spontaneous breathing test for 2 hours with the ventilator mode PS level set at 10 cm H_2_O and PEEP set at 5 cm H_2_O. Additionally, the patient had to meet the Wean Score criteria to be considered a successful SBT. Therefore, this study included model analysis based on the following inclusion criteria (Textbox 2).

**Inclusion and exclusion criteria.**

*Inclusion criteria*

- Pressure support (PS) level set at 10 cm H_2_O and positive end-expiratory pressure (PEEP) set at 5 cm H_2_O.

*Exclusion criteria*

- PS level greater or PS level less than 10 cm H_2_O, and PEEP greater or less than 5 cm H_2_O.

### Model Design

Given the constant advancements in technology, machine learning has now become widely used, primarily due to the effectiveness of information products. As a subset of data science, machine learning is now being further extended through the branch of deep learning. Therefore, we can generate models from extensive data through machine learning and develop predictions for various outcomes using existing data.

Deep learning is an algorithm centered within machine learning that focuses on representation learning from data. Several deep learning architectures exist, including deep belief networks, recurrent neural networks, and convolutional neural networks (CNNs). CNNs, a highly representative data-driven architecture, require large, high-quality datasets for effective model training. Data augmentation, transfer learning from pretrained models, and semi-automatic annotation are often used to address this issue. In the training phase of this study, multiple training datasets were prepared, each including the status feature inputs and corresponding outputs from various investigations of the study cases. CNNs succeed in image recognition and object detection because they can automatically extract spatial hierarchical features from images. Although this study does not include direct visual data, the characteristics of CNNs make it suitable for processing structured nonvisual data [34]. For instance, the convolutional layers of CNNs excel at capturing local patterns and correlations within the data, irrespective of whether these patterns come from spatial features in images, structural features in time-series data, or multidimensional matrices. We used a deep CNN to learn the features of older adults’ mental health before storing them in the network. Challenges in this process include preparing training data and designing learning algorithms. Subsequently, we tailor a learning algorithm suitable for the characteristics of the training data [35,36].

At the initial stage, the neural network is assigned random weight values, with its output differing significantly from the standard answer. After multiple learning iterations, the weights gradually adjust in the correct direction, resulting in a better or more accurate prediction. Given the multitude of feature factors influencing the prediction results of ventilator weaning, finding appropriate or correct features amongst the numerous characteristics is highly challenging.

**Figure 1:**
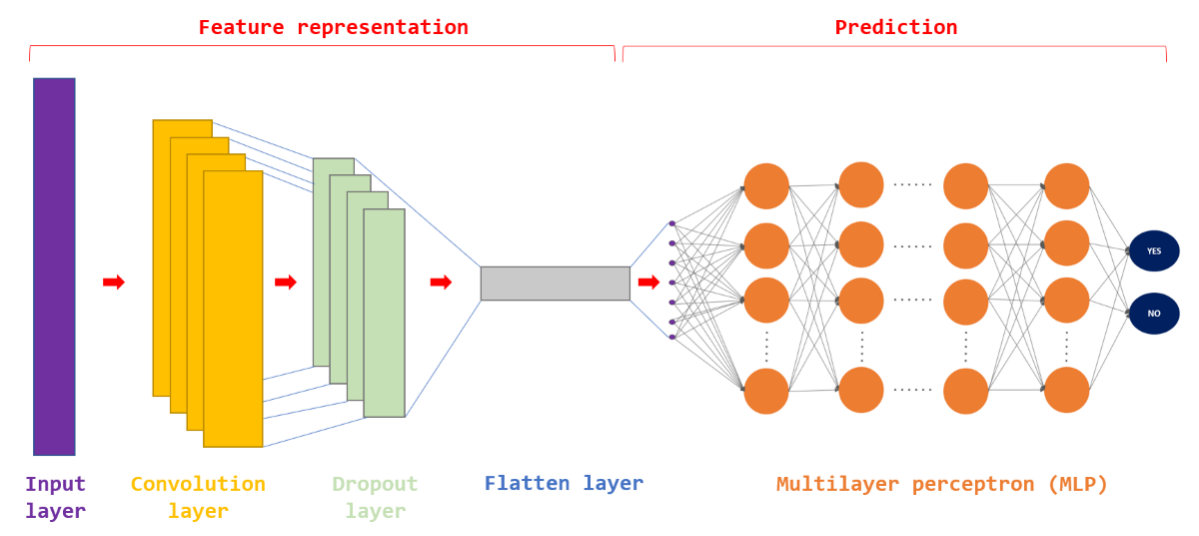
Architecture diagram of traditional CNN-MLP model. CNN: convolutional neural network.

Using the learning characteristics of deep learning, the model can autonomously deduce the characteristics of feature factors from the training data, thereby establishing a highly accurate prediction model. Figure 1 illustrates the architectural layout of the traditional CNN-MLP (convolutional neural network–multilayer perceptron) model. The model design begins with the input layer having the same number of neurons as the dimensions of the dataset. Subsequently, it connects to multiple convolutional layers, with pooling layers interspersed between them. Their primary function is to extract key features from the training data autonomously. Finally, the network concludes with a fully connected multilayer perceptron (MLP), transforming these features into 2 outputs. Selecting appropriate model initialization parameters is crucial for both the stability and convergence of training.

The optimization of the CNN model's ideal architecture is influenced by the task's complexity and the dataset's features. Since deep learning is primarily based on empirical learning methods, the efficiency of CNN models often stems from practical testing and iterative adjustments. The results of preliminary models can also serve as reference points for measuring performance improvements.

**Figure 2:**
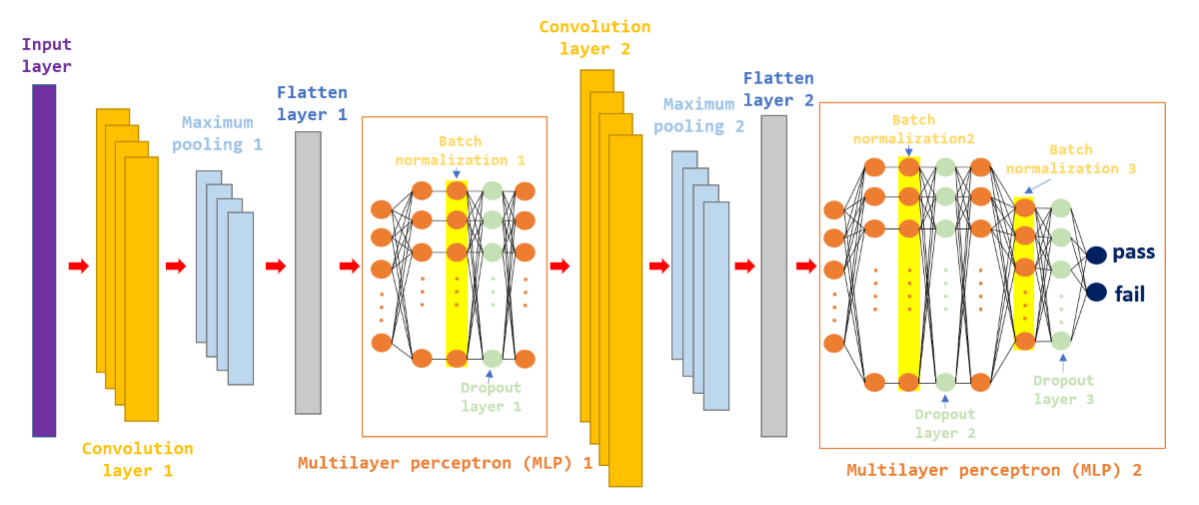
Architecture diagram of the hybrid CNN-MLP model. CNN: convolutional neural network.

In this study, we proposed a novel deep learning architecture, hybrid CNN-MLP, to predict the outcome of an SBT. Figure 2 depicts the proposed hybrid CNN-MLP model architecture in this study. Unlike the traditional CNN-MLP classification method, hybrid CNN-MLP performs feature learning and fusion by interleaving CNN and MLP layers so that data features can be extracted and integrated at different levels, thereby improving the flexibility and prediction accuracy of the model.

This study begins with a simple framework for the hybrid CNN-MLP design, gradually increasing its complexity by adjusting the number of layers and neurons. Starting with a straightforward structure accelerates training and evaluation, streamlining the continuous refinement of the hybrid CNN-MLP model. The hybrid CNN-MLP model architecture is explained as follows (Textbox 3).

**Hybrid CNN-MLP (convolutional neural network–multilayer perceptron) model architecture.**

The first CNN module

- Convolutional layer (14×64): the input channels are 14, the output channels are 64, and convolutional feature extraction is performed.
- Maximum pooling layer (kernel_size=2, stride=2): dimensionality reduction to retain the main features and reduce the amount of computation.

MLP (fully connected layer)

- Flatten layer: flatten the features extracted by CNN so that they can be input into the fully connected layer.
- Linear layer (64×128): fully connected layer with output dimensions of 128.
- Batch normalization layer (128): Stabilizes the training process.
- Dropout layer (*P*=.50): randomly inactivate 50% of neurons to prevent overfitting.
- Linear layer (128×128): fully connected, keeping the same dimension.

The second CNN module

- Convolutional layer (128×64): performs the convolution operation again.
- Maximum pooling layer (kernel_size=2, stride=2): reduces the dimension again to improve the generalization ability of the model.

The second phase of MLP

- Flatten layer: flatten into a vector for feeding into the MLP layer.
- Linear layer (64×256): fully connected layer with output dimensions of 256.
- Batch normalization layer (256): batch normalization to improve training stability.
- Dropout layer (*P*=.50): prevent overfitting.
- Linear layer (256×64): fully connected layer, reducing the dimension to 64.
- Batch normalization layer (64): batch normalization.
- Dropout layer (*P*=.30): reduce overfitting by inactivating 30% of neurons.
- Linear layer (64×2): output layer, the final output is 2-dimensional (probably representing a binary classification problem).

In the network used in this study, the Xavier Glorot initialization method [1] was used to assign weights. This method mitigates potential issues, particularly gradient vanishing or exploding problems in deep networks, by adjusting weights based on the quantity of input and output units. This ensures a smoother and more effective training process.

To avoid overfitting, we adopt multiple strategies to ensure the stability and generalization ability of the model. First, we use L2 regularization (weight decay) to discourage overly complex models from memorizing too much training data. Second, during the model training process, we applied the dropout technology to randomly deactivate some neurons to reduce the overadaptation (coadaptation) between neurons and improve the model's generalization ability. We also adopt the early stopping strategy to monitor the loss changes of the validation set during training. When the validation loss fails to decrease within several consecutive training cycles, training is stopped to prevent the model from overfitting to the training data. In addition, to ensure that the model is not too complex, we control the number of layers and neurons in the neural network to balance learning and generalization abilities.

Regarding hyperparameter tuning, we conducted multiple experiments to optimize the model performance. First, in terms of learning rate adjustment, we initially set it to 0.001 and used learning rate decay to gradually reduce the learning rate during training to improve convergence stability. For batch size, we tested different values, such as 32, 64, and 128, and finally chose 64 to balance training efficiency and model stability. Regarding the activation function, the hidden layer uses Rectified Linear Unit to solve the gradient disappearance problem, and the output layer uses Softmax for classification. For the optimizer, we tested Adam and Stochastic Gradient Descent and finally chose Adam because it performed best regarding convergence speed and stability. Finally, in terms of network architecture, we tested different numbers of layers and neuron configurations to ensure that the model can effectively learn data features while avoiding overfitting problems caused by excessive complexity.

During the training of the deep learning network model, this study inputs labeled data into the network, uses backpropagation and the Adam optimization algorithm, and adjusts the network weights based on the loss calculated using the binary cross-entropy function. On the other hand, the batch size used in this study is 64, with the model undergoing training for 6000 iterations. Of the dataset, 80% (5228/6536 records) is used as the training dataset, while the remaining 20% (1308/6536 records) is designated as the testing dataset to evaluate the model's performance on previously unseen data. Regular checkpoints and an early stopping mechanism ensure that the model used in this study does not overfit and retains its optimal performance architecture.

This study aims to develop a neural network model suitable for predicting respiratory outcomes. It aims to serve as an assessment standard and prediction tool for respiratory care, thus helping health care professionals provide higher quality health care. In establishing this study's intelligent respiratory care model, the prediction variable is based on the success or failure of the SBT, with relevant respiratory information incorporated into the model training.

### Evaluation Indexes

This study aims to develop an AI-based model to predict whether patients can successfully pass an SBT. Since SBT is a key test for whether mechanically ventilated patients can breathe spontaneously, and its prediction results directly affect clinical decision-making, it is crucial to evaluate the model's accuracy. This study adopted a variety of evaluation indicators to ensure the reliability and stability of the model in clinical applications.

When we try to predict whether a patient will successfully pass the SBT, accuracy alone may not be sufficient to evaluate the model's performance fully. Accuracy represents the proportion of all participants correctly predicted and is applicable when the proportion of SBT passes and fails is equal. However, if the data's ratio of successes to failures is unbalanced, focusing only on accuracy may lead to falsely optimistic estimates. For example, if most patients can successfully pass the SBT, and the model only predicts that “all patients will succeed,” although the accuracy may be high, it cannot effectively assist clinical decision-making. Therefore, we use precision and recall to evaluate the model's effectiveness.

Precision measures the proportion of patients predicted by the model to pass the SBT who did. This is crucial to avoid unnecessary SBTs. If the model's precision is too low, it means that among the successful cases it predicts, many patients actually cannot pass the SBT, which may lead to initiating SBT too early, resulting in respiratory muscle fatigue, hypoxemia, and even acute respiratory failure in patients. Therefore, a higher precision ensures that the model has a higher credibility when recommending the initiation of SBT.

Recall (also known as sensitivity) measures how many of the patients who successfully passed the SBT were correctly identified by the model. This has a critical impact on avoiding erroneous prolongation of mechanical ventilation. If the recall rate is too low, it means that many patients who should be able to be successfully weaned are incorrectly predicted to be unable to pass the SBT, causing them to continue to be maintained on mechanical ventilation, which may increase the risk of lung infection or muscle atrophy. Therefore, the higher recall ensures that no patients who can pass the SBT are missed.

In addition to precision and recall, this study also calculates the *F*_1_-score, which is a comprehensive indicator used to balance the trade-off between precision and recall. In clinical decision-making, we hope to ensure that the SBT is successfully passed (high precision) while not missing patients who should be able to pass the SBT (high recall), so the *F*_1_-score can be used as a measure of the overall effectiveness of the model.

Specificity is also an important evaluation indicator, measuring whether the model can correctly identify patients who cannot pass SBT. High specificity indicates that the model is effective in avoiding erroneous attempts to start SBTs in patients who cannot pass SBT. Finally, we also considered the false positive rate (FPR) and false negative rate (FNR) to analyze the model's error prediction pattern further and help clinicians understand the model's performance in different scenarios.

Based on the predictive results of the proposed model, we can obtain the following parameters: true positive, false positive, false negative, and true negative. Details of the evaluation metric formulas are available in Multimedia Appendix 1. The receiver operating characteristic (ROC) curve is an important tool for evaluating the classification ability of a model. It describes the changes in the true positive rate (TPR; recall rate) and FPR of the model at different decision thresholds. When the threshold is lower, the model is more likely to predict positive cases; TPR will increase, but FPR may also increase. When the threshold is higher, the model becomes more conservative, and FPR decreases, but some true positive cases may be missed. The closer the ROC curve is to the upper left corner, the better the classification ability of the model, while the ROC curve on the diagonal indicates that the classification effect of the model is no different from random guessing.

Area under the curve (AUC) is the area under the ROC curve, with a value range of 0-1, representing the overall classification ability of the model. The closer the AUC is to 1, the more stable the model is at different thresholds and the more effective it is in distinguishing different categories. In the SBT prediction model, a high AUC value means that the model can more accurately distinguish which patients can successfully pass the SBT and which patients still need mechanical ventilation support.

## Results

Table 1 presents the study cases’ demographic variables, respiratory parameters, and vital signs. Among the cases, the average age of patients is around 67.5 (SD 16.3) years. In the successful SBT group, the average age is 68.5 (SD 15.3) years, while in the failure group, it is 66.5 (SD 17.2) years. Regarding gender distribution, 4091 out of 6536 (62.6%) patients are male. Among them, 1178 of 3268 (36%) patients were in the successful SBT group, while 2913 of 3268 (89.1%) patients were in the failure group.

**Table 1:** Characteristics of intubated patients in the intensive care unit.

			Case group				
			Successful SBT^a^ (n=3268)		Failure of SBT (n=3268)		Total (N=6536)
**Demographic**							
Age (years), mean (SD)			68.5 (15.3)		66.5 (17.2)		67.5 (16.3)
**Gender, n (%)**							
	Male	1178 (36.0)		2913 (89.1)		4091 (62.6)	
	Female	2090 (64.0)		355 (10.9)		2445 (37.4)	
**Mechanical ventilator settings and respiratory function, mean (SD)**							
	FiO_2_^b^ (%)	32.9 (6.2)		37.5 (11.8)		35.2 (9.7)	
	PaO_2_^c^ (mm Hg)	107.4 (20.2)		109.6 (27.9)		108.5 (24.4)	
	VTe^d^ (exhaled VT; mL)	0.6 (1.7)		0.9 (2.9)		0.8 (2.4)	
	Measured RR^e^ (bpm)	19 (8)		21 (10)		20 (9)	
	Paw^f^ (cm H_2_O)	16.3 (1.9)		22.5 (5.2)		19.4 (5.0)	
	MAP^g^ (cm H_2_O)	8.3 (1.2)		11.3 (3.2)		9.8 (2.8)	
	MVe^h^ (exhaled MV; Lpm)	8.0 (3.0)		7.9 (2.4)		7.9 (2.7)	
**Vital signs, mean (SD)**							
	Heart rate (bpm)	84.9 (16.7)		87.6 (18.9)		86.2 (17.9)	
	Systolic blood pressure (mm Hg)	126.5 (20.2)		122.7 (22.5)		124.6 (21.5)	
	Diastolic blood pressure (mm Hg)	71.7 (14.4)		68.1 (15.4)		69.9 (15.0)	
	SpO_2_^i^ (%)	99 (2)		98 (3)		98 (2)	
	Body temperature (°C)	36.5 (0.5)		36.5 (0.6)		36.5 (0.5)	

^a^SBT: spontaneous breathing trial.

^b^FiO_2_: fraction of inspired oxygen.

^c^PaO_2_: partial pressure of oxygen.

^d^VTe: exhaled tidal volume.

^e^RR: respiratory rate.

^f^Paw: peak airway pressure.

^g^MAP: mean airway pressure.

^h^MVe: minute ventilation.

^i^SpO_2_: oxygen saturation.

For respiratory parameters, the average FiO_2_ is 35.2%, with the successful group at 32.9% and the failure group at 37.5%. The partial pressure of oxygen levels are similar across all groups, with slight variations. The failure group has a higher exhaled tidal volume and measured RR. The peak airway pressure and mean airway pressure are also higher in the failure group than in the successful group. Minute ventilation shows minimal difference between the groups.

Vital signs indicate that the heart rate is slightly higher in the failure group (87.6 bpm) compared to the successful group (84.9 bpm). Systolic and diastolic blood pressure are lower in the failure group. Oxygen saturation remains high across all groups, with minimal variation. Body temperature is consistent, averaging around 36.5 °C across all groups.

The PyTorch framework in Python is used to construct the proposed model.

**Figure 3:**
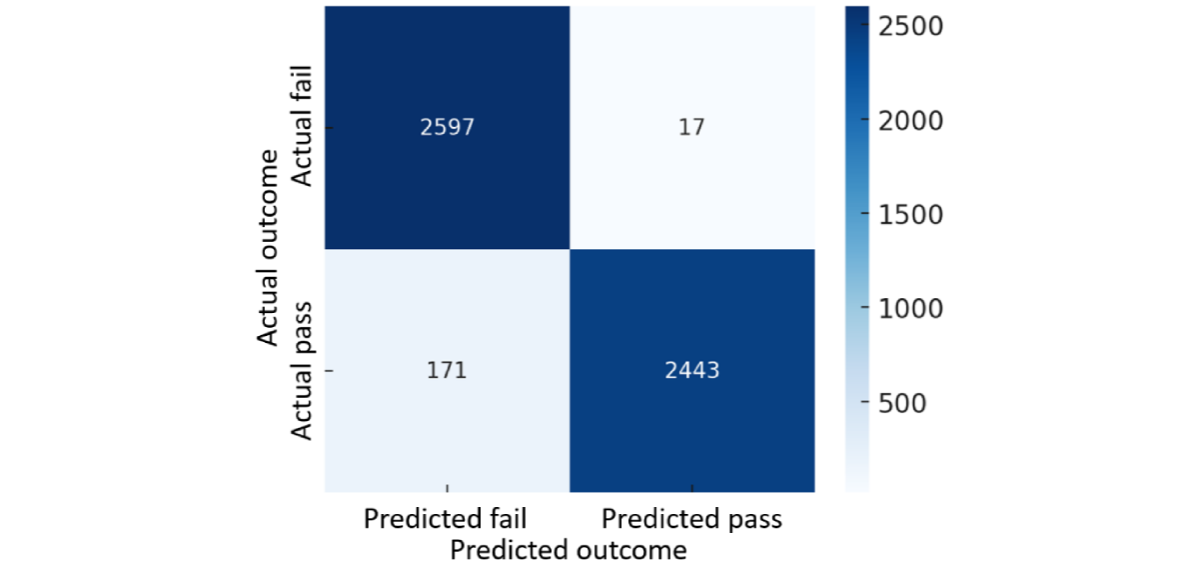
Confusion matrices for training data.

**Figure 4:**
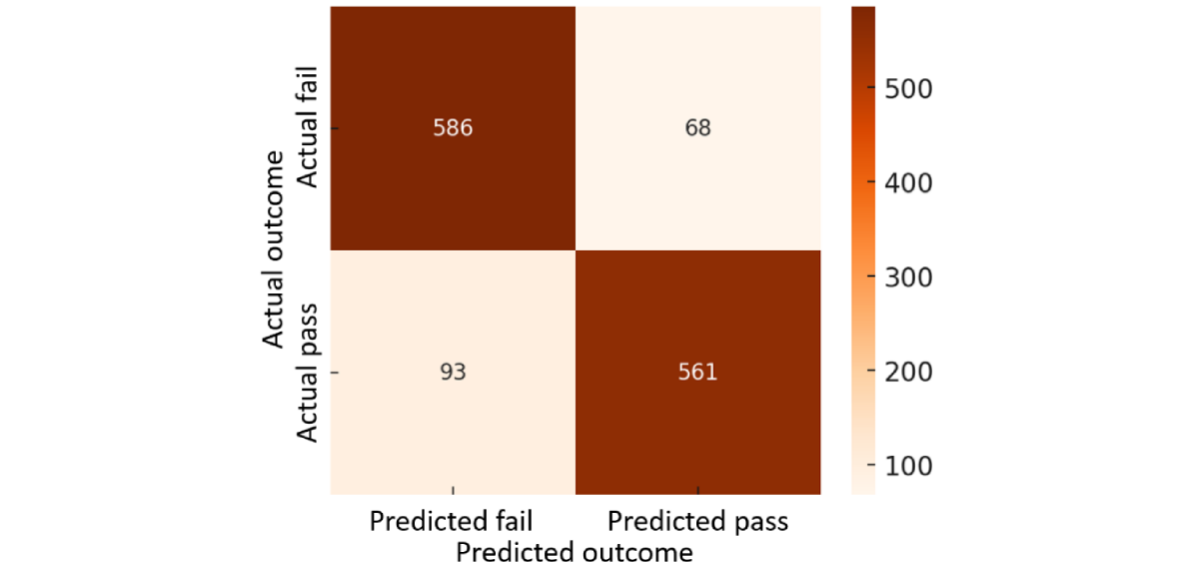
Confusion matrices for testing data.

In order to present the prediction results of the model more intuitively, this study uses the confusion matrix to show the classification of the model on the training and test datasets. The confusion matrix of the model’s prediction results is shown in Figures 3 and 4. The results show that the model performs well on the training data, and the classification ability on the test data remains stable. Although the number of misclassifications increases slightly, it is still within an acceptable range.

Table 2 illustrates the performance metrics of a model for predicting successful and failed SBT across both training and testing datasets.

**Table 2:** Performance metrics of the spontaneous breathing trial prediction model.

Metric	Training value	Testing value
Accuracy	96.4% (5040/5228)	87.7% (1147/1308)
Precision	99.3% (2443/2460)	89.2% (561/629)
Recall (sensitivity)	93.5% (2443/2614)	85.8% (561/654)
Specificity	99.3% (2597/2614)	89.6% (586/654)
FPR^a^	0.7% (17/2614)	10.4% (68/654)
FNR^b^	6.5% (171/2614)	14.2% (93/654)
*F*_1_-score	0.963	0.875

^a^FPR: false positive rate.

^b^FNR: false negative rate.

Overall, the model’s accuracy is 96.4% (5040/5228 records) in the training data and 87.7% (1147/1308 records) in the test data. Although the test results are slightly lower than the training results, they can still maintain a high accuracy, indicating that the model has good generalization ability and no overfitting problem.

Further analysis of the model's performance showed that the precision was 99.3% (2443/2460 records) in the training data and 89.2% (561/629 records) in the test data, indicating that the model had a slightly higher FPR on the test data but was still able to identify patients who could successfully pass an SBT effectively. In other words, in clinical applications, when the model predicts that a patient can pass the SBT, the likelihood of their actual success is still quite high, reducing the risk of unnecessary weaning failure.

However, in terms of recall (sensitivity), the recall rate in the training data was 93.5% (2443/2614 records), while it dropped to 85.8% (561/654 records) in the test data, indicating that some patients who should have passed the SBT in the test data were still not correctly identified. This may result in some patients being wrongly judged as unable to pass the SBT and continuing to receive unnecessary mechanical ventilation, thereby increasing the risk of lung infection or other complications.

In addition, the specificity was 99.3% (2597/2614 records) in the training data and 89.6% (586/654 records) in the test data, indicating that the model can still maintain good recognition ability for patients who cannot pass the SBT without significant deviation. This means that when the model predicts that a patient will not be able to pass the SBT, its judgment is correct in most cases, reducing the risk of the patient failing the SBT.

The *F*_1_-score, which is a comprehensive measure of precision and recall, is 0.963 in the training data and 0.875 in the test data, indicating that the model has achieved a certain balance between precision and recall and is suitable as a clinical decision-making aid.

**Figure 5:**
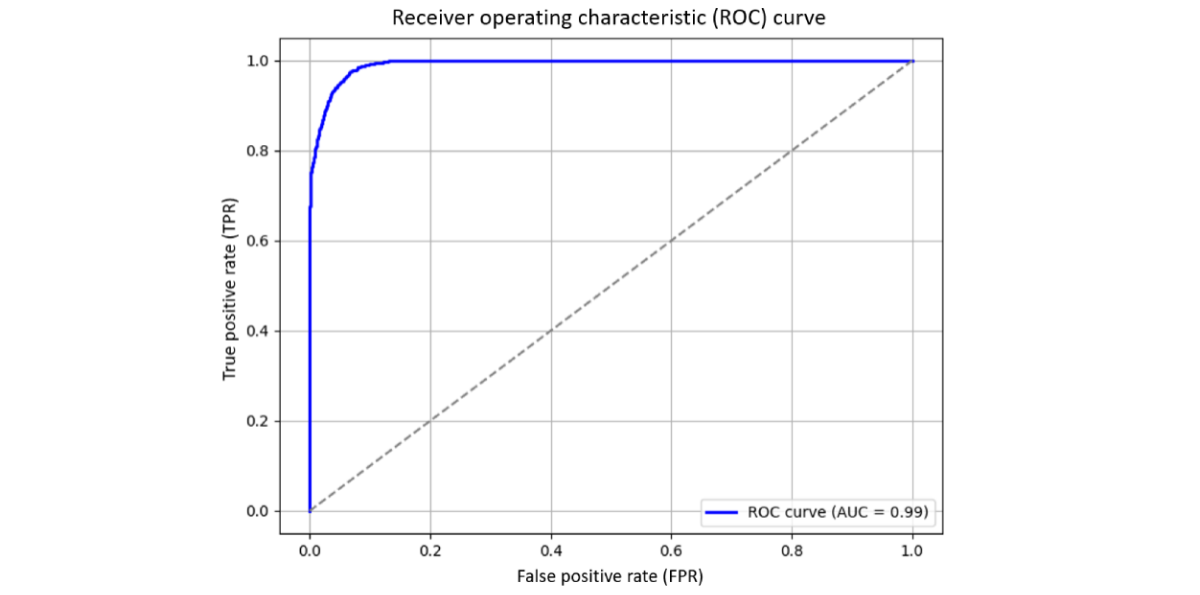
ROC curves for training data. AUC: area under the ROC curve.

**Figure 6:**
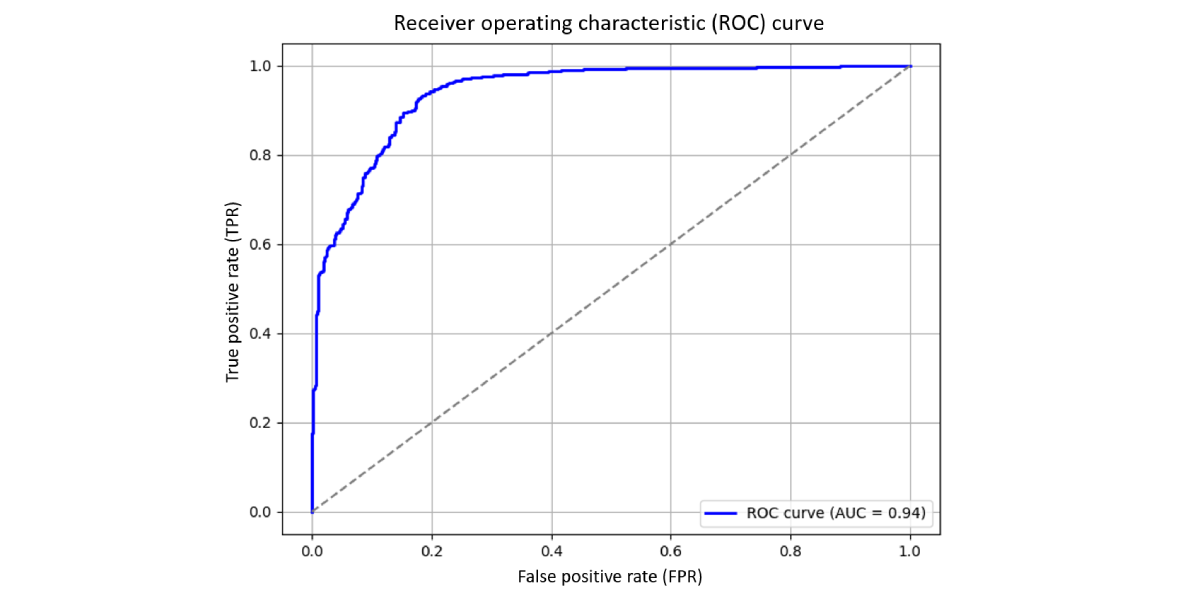
ROC curves for testing data. AUC: area under the ROC curve.

Figures 5 and 6 display the ROC curve of the SBT prediction model for both training and test data, with the AUC used to assess the model’s classification ability. Figure 5 is the ROC curve of the training data, and Figure 6 is the ROC curve of the test data. Both are used to measure the changes in the TPR and FPR of the model at different decision thresholds, and the AUC value is used to quantify the overall effectiveness of the model in distinguishing between successful and failed patients.

The results showed that the AUC for the training data was 0.99, indicating that the model could almost perfectly distinguish between successful and failed SBT patients during training, showing extremely high predictive ability. However, when the model was applied to the test data, the AUC dropped to 0.94, which is slightly lower than the training data but still within the excellent range, indicating that the model still has a good discrimination ability for unseen data. This means that although the model has a certain degree of generalization ability decline on the test data, it can still effectively distinguish which patients can successfully pass the SBT and which still need mechanical ventilation support.

This study successfully developed an AI-based SBT prediction model that can effectively assist clinicians in determining whether patients can successfully pass the SBT. Through evaluation metric analysis, including accuracy, precision, recall, specificity, *F*_1_-score, FPR, FNR, ROC curve, and AUC, we verified the robust performance of the model on both training and test data. Overall, the accuracy and reliability of the model in clinical applications have reached a high level and can provide strong support for clinical decision-making.

## Discussion

### Overview

This study demonstrates the potential of deep learning technology in evaluating SBT, which can effectively improve the accuracy and efficiency of managing mechanically ventilated patients. Traditional SBT assessment methods rely on simple threshold settings, which cannot fully reflect the patient's complex physiological state. The deep learning model developed in this study can integrate multidimensional clinical data to provide more precise prediction results that conform to actual physiological conditions.

A major innovation of this study is the design of the hybrid CNN-MLP architecture. Compared with the traditional deep learning model that only relies on CNN for feature extraction and MLP for classification, this study uses CNN and MLP layers alternately to form a novel feature learning and fusion method. This design helps capture features at different levels, making the model more flexible and efficient when processing data with complex feature structures. The research results show that the model achieves high accuracy in the training phase and maintains stable performance in the testing phase. The final *F*_1_-score on the test dataset is about 0.875, which proves its feasibility and reliability in predicting SBT results. These findings are consistent with previous studies [37,38], showing that AI technology can improve clinical decision-making accuracy and patient outcomes in critically ill patient care.

This study aimed to establish a model to predict whether a patient can successfully pass an SBT. The data division does not necessarily lead to data leakage in our research setting because our model learns the relationship between pre-SBT clinical parameters and SBT outcomes. That means the model does not track a patient's historical SBT trials but makes independent predictions based on the patient's current state before each SBT trial. We treat each SBT trial as an independent sample rather than linking multiple records from the same patient. Even if a patient has undergone multiple SBTs at different time points, each trial is considered a separate instance for training and prediction. Therefore, within-patient clustering does not significantly impact the model's learning process, and even if records of the same patient at different time points appear in the training and test sets, the model still learns the pattern that determines a patient's likelihood of passing the SBT, rather than focusing on individual patient characteristics.

Regarding patients who have undergone multiple SBT attempts with varying outcomes, the model learns to predict each SBT outcome independently based on pre-SBT clinical records of that specific attempt rather than being influenced by previous trials. Therefore, it does not distinguish between patients who succeeded on their first attempt and those who required multiple attempts; instead, it evaluates each instance based on its pre-SBT clinical record.

This study did not specifically adjust the time variables since the time factor has a limited impact on the model based on the following considerations. The SBT implementation standards of the research institutions were consistent with the clinical operation procedures throughout the study period. Therefore, data from different years can be regarded as homogeneous and will not significantly impact the model training and prediction results. In addition, during the data partitioning process, we used random sampling to ensure that the data distribution between the training and test sets was balanced to reduce the possible impact of time variability.

### Strengths and Limitations

The innovation of this study is to develop an AI-based prediction model to evaluate the outcome of an SBT under a specific SBT strategy using clinical parameters collected before an SBT begins. Traditionally, clinicians must comprehensively assess multiple physiological parameters to determine whether a patient is suitable for SBT. However, this process is highly subjective, experience-dependent, and has a high risk of misjudgment. This study uses AI technology to develop an objective and efficient prediction model to reduce the error of traditional manual evaluation and improve the accuracy of predicting whether an SBT will be successful.

The model in this study is built on pre-SBT clinical data to identify the relationship between these variables and SBT outcomes and to make predictions under a fixed SBT strategy. Unlike traditional approaches, this study does not compare different SBT strategies or analyze their impact on extubation success rates. Instead, it focuses on developing a data-driven method within a specific SBT strategy's framework to accurately predict whether patients can successfully pass SBT, providing clinicians with an objective and reliable decision-making aid.

Furthermore, the AI prediction model in this study focuses on predicting SBT success and highlights the clinical value of identifying patients who may fail SBT in advance. In current clinical practice, the SBT evaluation process typically takes 48-72 hours, and initiating SBT too early can result in respiratory muscle fatigue, hypoxemia, and even acute respiratory failure in patients. The proposed model can predict whether an SBT will be successful in advance and assist clinicians in identifying patients who may not be ready yet, which would prevent unnecessary testing, reduce the physiological burden on patients, and enhance the efficiency of ICU resource management.

The main advantage of this study lies in its innovative CNN-MLP hybrid architecture, which enables the model to effectively learn and fuse feature information at different levels and improve the prediction ability of SBT results. Compared with traditional deep learning models, the method of this study can not only extract features from image and time series data through CNN but also further enhance the understanding of high-dimensional features through MLP, thereby improving the accuracy and applicability of the model. In addition, by integrating multidimensional physiological data, the model of this study can more comprehensively assess the patient's clinical condition and provide more accurate and personalized extubation decision support compared with traditional threshold-based indicators.

The final *F*_1_-score on the test dataset is about 0.875, which proves its feasibility and reliability in predicting SBT results. The AUC for the test data was 0.94, indicating that the model has a good discrimination ability for unseen data. The study showed that the model maintained stable predictive performance during the testing phase and exhibited an excellent *F*_1_-score, confirming its potential in clinical applications.

The contributions of this study are summarized as follows:

1. Enhancing clinical decision support and prediction accuracy: Traditionally, clinicians assess whether patients are suitable for SBT by comprehensively analyzing multiple physiological parameters. This process relies heavily on experience and is subjective, which can easily lead to errors in judgment. This study uses AI technology to develop a prediction model based on pre-SBT clinical data, providing more accurate prediction results, reducing manual evaluation errors, and enhancing SBT prediction accuracy.
2. Identifying patients who may fail in advance: In current clinical practice, SBT evaluation typically takes 48-72 hours. If SBT is conducted too early, it may lead to respiratory muscle fatigue, hypoxemia, and even acute respiratory failure in patients. The AI prediction model introduced in this study can identify patients who may not successfully pass the SBT beforehand, preventing unnecessary trials, reducing the physiological burden on patients, and effectively managing ICU resources to enhance overall medical efficiency.
3. Innovative hybrid CNN-MLP model design: This study's methodological innovation proposes a hybrid deep learning architecture called hybrid CNN-MLP. This architecture differs from the traditional CNN-MLP classification method since it uses a unique approach to feature learning and fusion through the interlaced combination of CNN and MLP layers, where the data features can be extracted and integrated at various levels, making the model more flexible and efficient in processing complex data structures and enhancing the accuracy and applicability of SBT predictions.

However, this study still has some limitations. First, the data in this study came from a single medical institution, which may affect the model's generalization ability and limit its applicability in different clinical settings. Therefore, its robustness needs to be verified through multicenter data in the future. Second, although the model shows high accuracy at the technical level, in practical application, it is still necessary to consider how to integrate it with the clinical workflow to ensure that medical staff can adopt the technology smoothly. In addition, in the test dataset, the model’s performance decreased slightly compared to the training stage, indicating that there is still room for further optimization. These challenges can be overcome in the future through multicenter data expansion, improved model training strategies, and enhanced AI interpretation capabilities.

### Future Perspectives

In order to enhance the influence and application value of this study, the data scope should be further expanded in the future, and model training and verification should be carried out with multicenter data to ensure its applicability in different medical institutions and diverse patient groups. By introducing data from different medical environments, not only can the model's generalization ability be increased, but its adaptability to patients from different groups can also be improved. In addition, prospective clinical trials should be conducted to evaluate the impact of this model in actual clinical decision-making, such as whether it can effectively reduce the duration of mechanical ventilation, improve the success rate of extubation, and further analyze its impact on the clinical prognosis of patients.

In addition to increasing the breadth of data and validating real-world applications, the model in this study can be further optimized to improve its performance and interpretability. Although the deep learning model used in this study has strong predictive capabilities, it is a “black box model” with low interpretability. Hence, to ensure that the model applies to different health care systems, we pay special attention to the availability and variability of the input features. The characteristics used in this study are key indicators in clinical diagnosis and monitoring. These variables were selected primarily based on clinical relevance and availability. However, there may be variations in equipment and data collection methods between different medical systems. For example, the type of ventilator or blood oxygen monitor used in different wards may affect the measurement of certain values. Therefore, when the model is applied to different medical institutions in the future, it is recommended to first conduct a feature distribution analysis to evaluate the input data's adaptability and reduce heterogeneity's impact on model performance through transfer learning or regularization techniques.

By improving the model's training strategy, such as applying transfer learning or reinforcement learning, the model can be helped to learn feature patterns from different environments more effectively, thereby improving its adaptability. In addition, the interpretability of the model is also an important direction for future development. By designing a more transparent AI model architecture, medical staff can understand the decision-making logic of the model, which will help enhance clinical users' trust in AI-assisted decision-making systems and further promote their application in the medical field.

Finally, future attention should be paid to the model's actual deployment and clinical integration and the possibility of embedding it into the EHR system. Through seamless integration with existing medical information systems, the model can be ensured to be immediately available in clinical decision-making and enhance its auxiliary value to medical staff. In addition, when designing the user interface, the user-centered design principles should be considered so that medical staff can operate and understand the system more intuitively, ensuring that it can truly become an effective tool in the clinical decision-making. Through these efforts, deep learning–based SBT assessment technology will be able to be more widely used in clinical fields, improve the efficiency of mechanical ventilation management, and ultimately improve patients' treatment and rehabilitation outcomes.

### Conclusions

This study demonstrates the potential of deep learning technology to predict whether a patient can successfully pass an SBT, improving the accuracy and efficiency of mechanically ventilated patient management. Traditional SBT evaluation methods rely on simple threshold settings and are difficult to fully reflect the patient's complex physiological state. This study's AI prediction model integrates multidimensional clinical data to provide more accurate prediction results that meet actual clinical needs.

The main innovation of this study is the proposed hybrid CNN-MLP architecture, which uses CNN and MLP layers alternately for feature learning and fusion. Compared with traditional deep learning models, this method can more flexibly capture features at different levels and improve the model's ability to handle complex data structures. The results showed that the model achieved an *F*_1_-score of 0.875 on the test dataset, confirming its feasibility and reliability in predicting the success of SBT, which is consistent with previous research results on the application of AI technology in critical care.

In addition, this study highlights the value of AI prediction models in clinical decision support, especially in identifying patients who may not pass the SBT in advance, thereby reducing unnecessary trials, reducing the physiological burden on patients, and improving the management efficiency of ICU resources. However, the study's limitations are that the research data came from a single medical institution and, as a consequence, may affect the generalization ability of the model. In the future, it should be verified through multicenter data. In addition, integrating this technology seamlessly into clinical workflow and increasing the acceptance of medical staff are challenges that need to be addressed in the future.

In the future, this study recommends further expanding the data scope and evaluating the model's applicability in different medical institutions and patient groups through multicenter clinical trials. In addition, the model can be optimized through transfer learning or reinforcement learning to make it more adaptable to data patterns in different environments. Besides, the model's interpretability will be improved to enhance the trust of medical staff, and its integration into the EHR system will be explored to ensure that it can provide real-time support for clinical decision-making.

In summary, the hybrid CNN-MLP proposed in this study can improve the accuracy and efficiency of SBT assessment and provide a more reliable auxiliary tool for clinical decision-making. The ultimate goal is to optimize the mechanical ventilation weaning process, improve patient prognosis, and enhance the quality of ICU care.

## Appendix

Multimedia Appendix 1 Detailed mathematical formulas used in model construction and evaluation.

## Abbreviations

AI: artificial intelligence
AUC: area under the curve
CNN: convolutional neural network
CPAP: continuous positive airway pressure
EHR: electronic health record
FiO2: fraction of inspired oxygen
FNR: false negative rate
FPR: false positive rate
ICU: intensive care unit
IRB: institutional review board
MLP: multilayer perceptron
PEEP: positive end-expiratory pressure
PS: pressure support
ROC: receiver operating characteristic
RR: respiratory rate
SBT: spontaneous breathing trial
TPR: true positive rate
