# Supplementary material for: Prediction of Spontaneous Breathing Trial Outcome in Critically Ill-Ventilated Patients Using Deep Learning: Development and Verification Study
Source: JMIR Med Inform. 2025 May 21;13:e64592. doi: 10.2196/64592 (PMC12138301; doi:10.2196/64592)
Supplement: Multimedia Appendix 1 [file medinform_v13i1e64592_app1.docx]

## Multimedia Appendix 1

Based on the predictive results of the proposed model, we can obtain the following parameters: true positive (TP), false positive (FP), false negative (FN), and true negative (TN). The formulas used for the evaluation metrics presented in this study are as follows.

$Accuracy=\frac{TP+TN}{TP+TN+FP+FN}$, (1)

$Precision=\frac{TP}{TP+FP}$, (2)

$Recall=\frac{TP}{TP+FN}$, (3)

$Specificity=\frac{TN}{FP+TN}$, (4)

$F1 Score=\frac{2 \times Precision \times Recall}{Precision + Recall}$, (5)

$FPR=\frac{FP}{FP+TN}$, (6)

$FNR=\frac{FN}{FN+TP}$. (7)
